# Supplementary material for: Structural and Functional Insights into Dishevelled-Mediated Wnt Signaling
Source: Cells. 2024 Nov 11;13(22):1870. doi: 10.3390/cells13221870 (PMC11592973; doi:10.3390/cells13221870)
Supplement: Supplementary file 1 [file cells-13-01870-s001.zip › cells-3285960-supplementary.pdf]

Supporting Information (SI)

Structural and Functional Insights into Dishevelled-Mediated Wnt Signaling

Lei Wang [1], Rui Zhu [1], Zehua Wen [1], Hua-Jun Shawn Fan [1], Teresa Norwood-Jackson [2], Danielle Jathan [2] and Ho-Jin Lee [2,*]

[1] College of Chemical Engineering, Sichuan University of Science and Engineering, Zigong 643000, China; 322086001105@stu.suse.edu.cn (L.W.); zhurui0723@icloud.com (R.Z.); zehuawen5733@163.com (Z.W.); fan27713@yahoo.com (H.-J.S.F.)

[2] Division of Natural & Mathematical Sciences, LeMoyne-Owen College, Memphis, TN 38126, USA; tnorwood_jackson295@loc.edu (T.N.-J.); djathan240@loc.edu (D.J.)

* Correspondence: ho-jin_lee@loc.edu

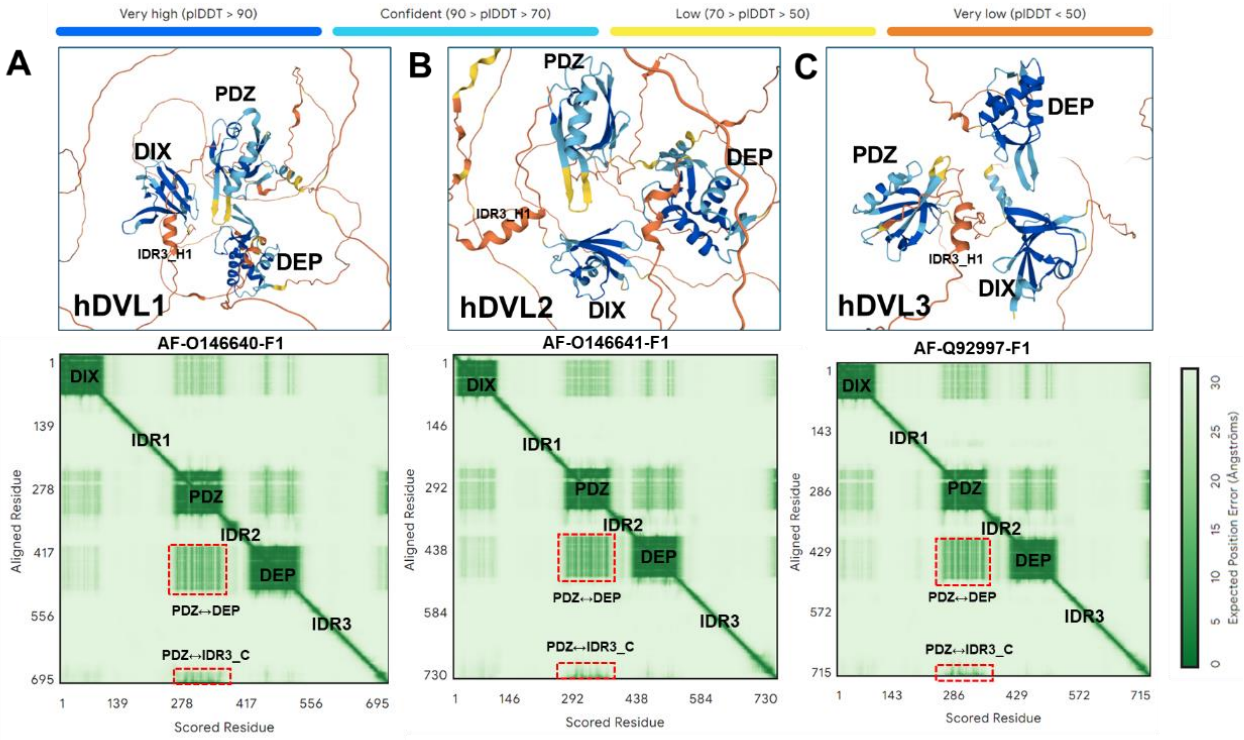

Figure S1. AF3-predicted DVL proteins (**A**) human DVL1, (**B**) DVL2, and (**C**) DVL3. The autoinhibited DVL proteins predicted by AlphaFold can be downloaded from AlphaFold Protein Structure Databases (hDVL1, AF-O146640-F1, hDVL2, AF-O146641-F1, and hDVL3, AF-Q92997-F1). The color code of pLDDT values is shown on the top. The PAE map for each predicted DVL protein is also shown.

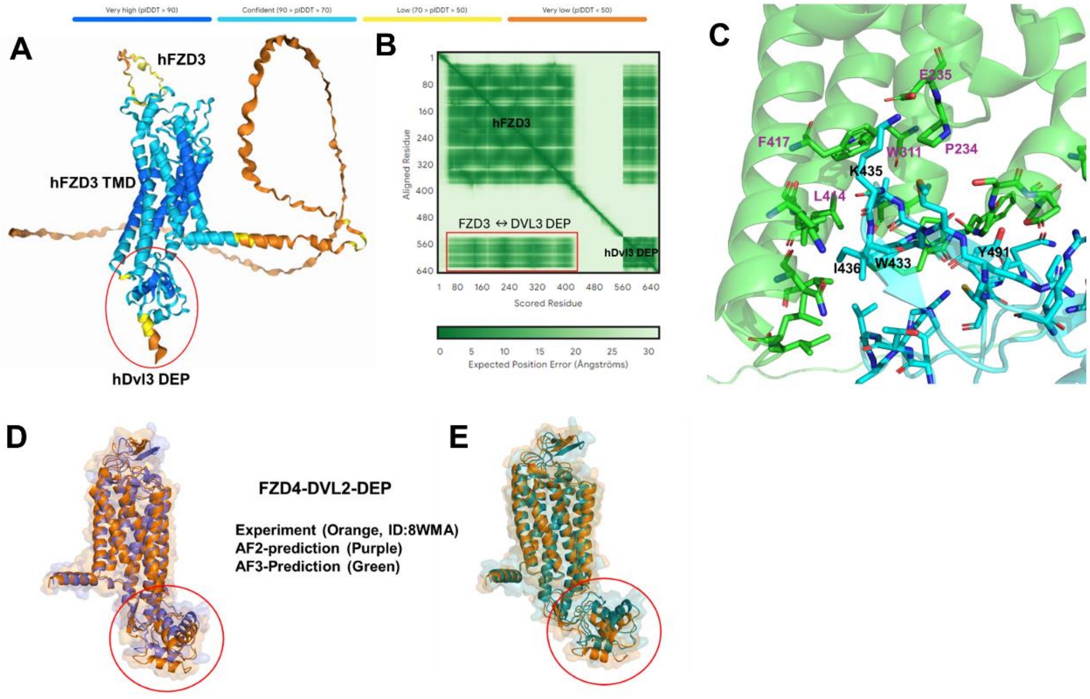

Protein Complex	AF2-EX	AF3-EX
RMSD	1.599	1.085

Figure S2. The interaction between the hFZD transmembrane domain (TMD) and the hDVL DEP domain can be predicted by AF2 (or AF3). (**A**) AF3-predicted hFZD3 TMD structure in the complex with the hDVL3 DEP domain. The color code of pLDDT values is shown on the top. (**B**) the PAE map of AF3-predicted complex structure of hFZD3 TMD and hDVL3 DEP. (**C**) The interfacing residues of hFZD3 TMD and hDVL3 DEP complex structure predicted by AF3. (**D** and **E**) The AF3-predicted complex structure is better than the AF2-predicted one. Comparison of predicted structures from (**D**) AF2 (https://github.com/sokrypton/ColabFold, accessed on 9 June 2024) and (**E**) AF3 (https://alphafoldserver.com, accessed on 6 Sept. 2024) with an experimentally determined structure from cryo-EM experiment of the complex between hFZD4 and hDVL2 DEP (ID: 8WMA).

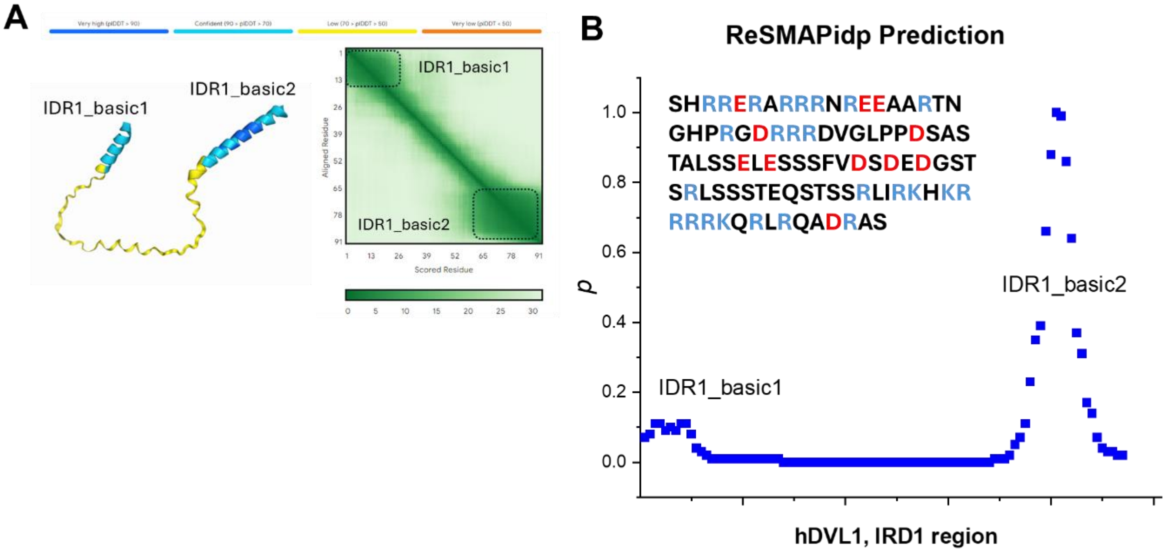

Figure S3. AF3-predicted the Intrinsic disordered region (IDR) 2 fragment of human DVL1 protein. (A) AF3 predicts IDR1_basic1 and IDR2_basic2 may form putative helices. (B) ReSMAPidp predicts IDR1_basic2 may bind to the membrane. The sequence of hDVL1 is shown. The positive residues (blue) and the negative residue (red) are shown.

Table S1. Analysis of interface, solvent-accessible area, hydrogen bond, and salt bridges of the complex structure of hFZD3 TMD and hDVL3 DEP. [a]

AF3-Predicted Structure	hFZD3 TMD	hDVL3 DEP
**Number of Residues**		
Interface	27 (5.0%)	20 (19.4%)
Surface	512 (95.2%)	91 (88.3%)
Total	538 (100.0%)	103 (100.0%)
**Solvent-accessible Area, A**		
Interface	761.8 (1.8%)	868.1 (12.9%)
Total	42424.1 (100%)	6720.7 (100%)
Hydrogen Bonds (Distance, Å)		
3.39	S321 [OG]	M432[O]
2.80	G320 [N]	Q489 [O]
2.97	S321 [N]	Y491 [OH]
3.84	E322 [N]	Y491 [OH]
2.61	E235 [OE2]	K435 [NZ]
2.97	A314 [O]	W433[NE1]
3.16	P317 [O]	K483 [NZ]
3.18	P317 [O]	C490 [SG]
3.31	K318 [O]	C490 [SG]
3.05	E322 [OE1]	R429 [NH2]
3.34	E322 [OE1]	R429 [NH1]
Salt Bridges (Distance, Å)		
3.04	E235 [OE1]	K435[NZ]
2.61	E235 [OE2]	K435 [NZ]
3.05	E322 [OE1]	R429 [NH2]
3.34	E322 [OE1]	R431 [NH1]

[a.] PDBePISA web server [174,175] was used to analyze the interface between hFZD3 TMD and hDVL3 DEP (accessed on 31 Oct. 2024)

Table S2. DVL PDZ inhibitors (small molecules) were reported in the literature.

Compounds	Mechanism	Refs
FJ9	Down-regulation of canonical Wnt signaling and inhibition of tumor cell growth by disrupting the interaction between the Frizzed-7 Wnt receptor and the Dvl PDZ domain.	[71]
3289-8625	Competitively inhibits the Wnt signaling pathway at the Frizzed-Dvl interaction site.	[72]
Sulindac	Blocking typical Wnt signaling downstream of the DVL PDZ domain.	[48]
J01-017a	Competes for binding to the Dapper peptide, a DVL PDZ domain binding peptide	[80]
KY-02061, KY-02327	KY-02061 interacts with the Dvl PDZ domain similarly to DBM (Dvl binding motif) and enhances osteoblast differentiation; KY-02327, a metabolically stable KY-02061 analog, interacts with the Dvl PDZ domain similarly to KY-02061, activates the Wnt/β-catenin signaling pathway and promotes osteoblast differentiation.	[176]
BMD4702	Directly binding ligand binding sites of the DVL PDZ domain.	[172]
NPL-4011	NPL-4011 is a stronger inhibitor than compound 3289-8625 when compared under identical conditions using hDvl1PDZ	[177]
RS4690	Computational studies led to the discovery of racemate RS4690, showing selective inhibition of DVL1 binding.	[178]
